# Immunotherapy against esophageal primary amelanotic malignant melanoma relapse

**DOI:** 10.1093/jscr/rjab393

**Published:** 2021-10-13

**Authors:** Ryoichi Tsukamoto, Hiroaki Ihara, Masaru Takase, Ai Shimazu, Masahiko Takei, Hiroyoshi Miura, Kazuhiro Sakamoto, Koji Namekata

**Affiliations:** Department of Coloproctological Surgery, Juntendo University, Faculty of Medicine, Tokyo, Japan; Department of Respiratory Medicine, Juntendo University, Faculty of Medicine, Tokyo, Japan; Koto Hospital, Tokyo, Japan; Department of Pathology, Koshigaya Municipal Hospital, Saitama, Japan; Department of Surgery, Koshigaya Municipal Hospital, Saitama, Japan; Department of Surgery, Koshigaya Municipal Hospital, Saitama, Japan; Department of Surgery, Koshigaya Municipal Hospital, Saitama, Japan; Department of Coloproctological Surgery, Juntendo University, Faculty of Medicine, Tokyo, Japan; Department of Surgery, Koshigaya Municipal Hospital, Saitama, Japan

## Abstract

Melanoma is a malignant tumor derived from melanocytes. Esophageal melanomas occur infrequently, especially primary amelanotic malignant melanoma of the esophagus (PAMME), which is extremely rare. Here, we report the case of a 74-year-old man with an esophageal amelanotic melanoma on the esophagogastric junction (EGJ) found on esophagogastroduodenoscopy. Radical surgery for the tumor at the EGJ was performed with total gastrectomy and D2 lymph node dissection. Diagnosis of PAMME was confirmed postoperatively by immunohistochemical staining. Four months after the surgery, abdominal computed tomography revealed multiple liver metastases. The patient received seven cycles of nivolumab monotherapy and two subsequent cycles of nivolumab and ipilimumab, and these metastases diminished. Recently, new therapeutic agents including immunotherapy have been developed for malignant melanoma and these agents have the potential of improving the prognosis of PAMME. Here, we present new insights into the diagnosis and therapeutic methods that can be used against primary esophageal melanoma.

## INTRODUCTION

Melanoma is a malignant tumor derived from melanocytes. More than 90% of malignant melanomas are of cutaneous origin and only 4–5% are extracutaneous. The incidence of melanoma has been steadily increasing in the past several decades [1, 2]. Surgical excision is curative in most cases of early-stage malignant melanoma; however, the management of disseminated disease remains complicated. Recently, new therapeutic options such as checkpoint inhibitor immunotherapies and molecular targeted therapies for the treatment of cutaneous melanomas have become available [3, 4], but the evidence against extracutaneous melanomas is scarce. Esophagogastrointestinal melanomas occur infrequently [5] and are predominantly diagnosed in the rectum and anus; therefore, primary amelanotic malignant melanoma of the esophagus (PAMME) is a relatively rare entity. Because there is no established standard treatment for PAMME, the efficacy of chemotherapy in improving the prognosis is uncertain for the patients with advanced disease.

We herein report a case of PAMME forming an ulcerated protuberant mass on the esophagogastric junction (EGJ).

## CASE REPORT

**Figure 1:**
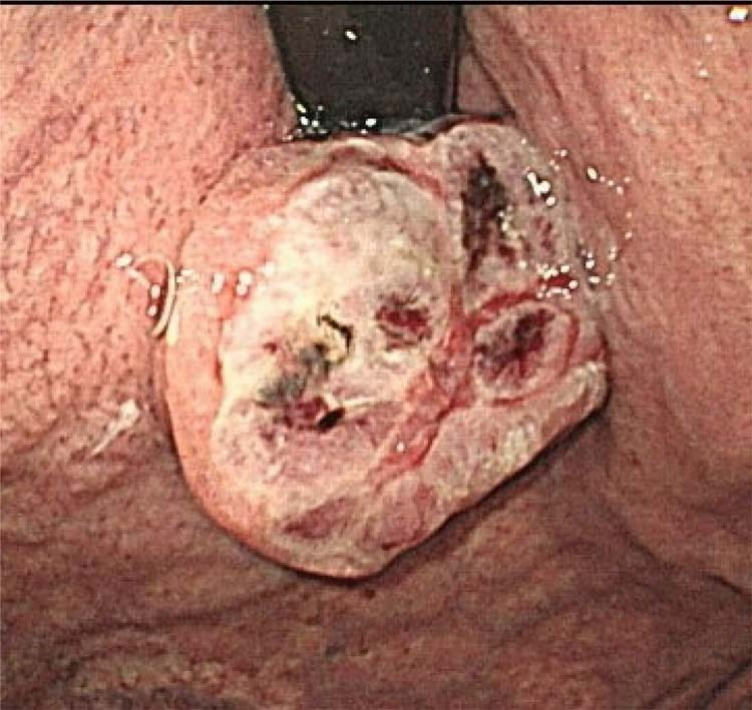
Endoscopic examination showing a large tumor with an ulcer just adjacent to the EGJ at the cardia.

**Figure 2:**
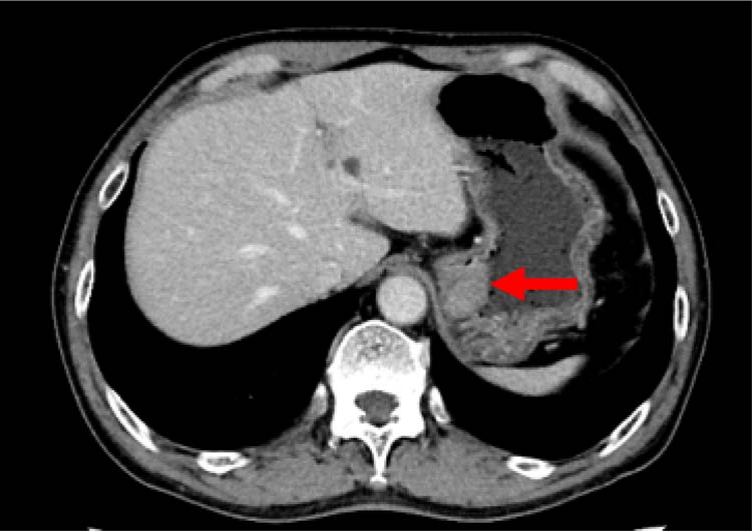
Contrast-enhanced CT showing the thickness of the gastric wall in the upper stomach (red arrow).

**Figure 3:**
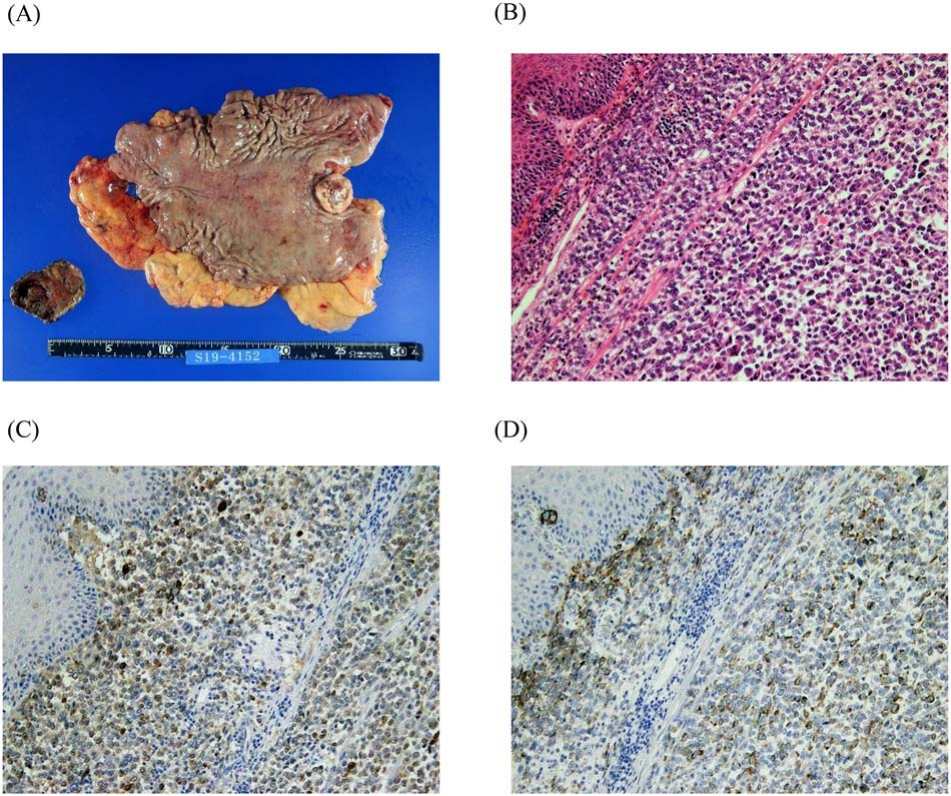
Macro-photograph and histopathological analyses of the surgical specimen. A whitish amelanotic mass with central ulceration at the EGJ and protrusion into the gastric lumen. (macro-photograph) (**A**). Polygonal and round neoplastic cells with round nuclei and prominent nucleoli nestled within the esophageal squamous epithelium (H&E staining, ×200) (**B**). The cells were positive for HMB-45 and S-100, respectively (HMB-45 and S-100 IHC staining, ×200) (**C** and **D**).

**Figure 4:**
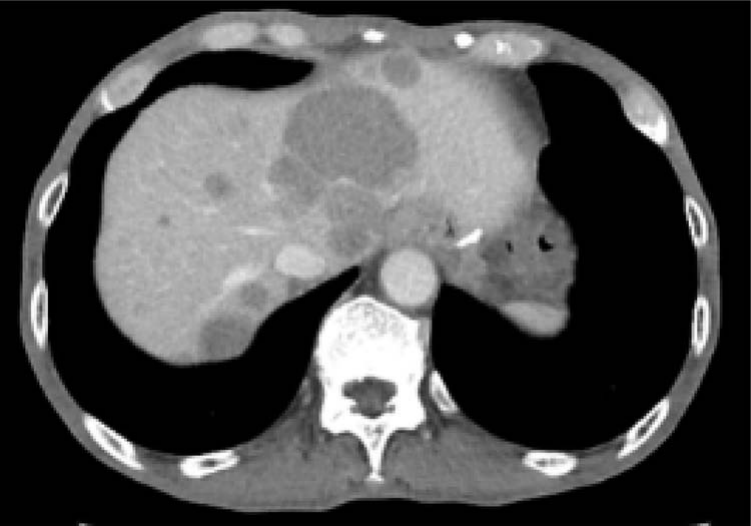
Contrast-enhanced CT showing progression of multiple cystic metastases in the liver.

A 74-year-old man presented to the hospital for follow-up esophagogastroduodenoscopy (EGD) after *Helicobacter pylori* eradication. EGD revealed a large necrotic ulcerated mass at the cardia of the stomach (Fig. 1). Endoscopic biopsy revealed a malignant tumor negative for cytokeratin AE1/AE3, CD45, chromogranin A and synaptophysin on immunohistochemical (IHC) staining, and initial diagnosis was poorly differentiated adenocarcinoma. Physical examination, routine blood investigations and serum tumor marker levels, including carcinoembryonic antigen, CA-125 and CA 19-9, were normal. Abdominal computed tomography (CT) showed thickness of the stomach wall directly below the esophagus (Fig. 2, red arrow). Enlarged lymph nodes and distant metastatic lesions were absent. Radical surgery including total gastrectomy and D2 lymph node dissection was performed. Surgical specimens demonstrated a whitish amelanotic mass with central ulceration, present at EGJ, measuring 38 mm × 30 mm and protruding into the gastric lumen (Fig. 3A). Histological examination revealed polygonal and round neoplastic cells with round nuclei and prominent nucleoli nestled within the esophageal squamous epithelium, thus forming an ‘*in situ*’ lesion. No melanin deposition was found in the neoplastic cells. Immunohistochemically, neoplastic cells were positive for HMB45 and S-100 (melanoma markers; Fig. 3B–D). The final diagnosis was primary amelanotic malignant melanoma of esophagus, T2, N0 and M0 (UICC [International Union against Cancer] classification system, 7th ed.). Four months after surgery, abdominal CT revealed multiple liver metastases. The patient received seven cycles of nivolumab monotherapy, with two subsequent cycles of nivolumab and ipilimumab. Liver metastases were initially controlled by immunotherapy, but they eventually worsened, as assessed by the Response Evaluation Criteria in Solid Tumors ver. 1.1 (Fig. 4).

## DISCUSSION

The vast majority of esophagogastrointestinal melanomas are metastases from cutaneous melanomas. Melanocytes are present in the oral, esophageal and anorectal mucosa. PAMME is rare with an estimated frequency of 0.1–0.2% of all the malignancies arising in the esophagus [6], with the lower and middle thirds of the esophagus being the most common locations due to a greater concentration of melanocytes [7]. Dysphagia is a common presenting symptom. Owing to the soft nature of the tumor, fewer symptoms are observed than those for esophageal carcinoma. Therefore, PAMME is often diagnosed at advanced stages, with ~30–40% of the patients having lymph node involvement or distant metastases [8]. Metastasis from other sites must be ruled out before making a diagnosis of PAMME. In our case, no primary lesion on the skin, mucosa or any other organ was discovered; the final diagnosis was PAMME. An accurate diagnosis is very important, as the prognosis of PAMME is more favorable than that of metastatic melanoma [9]. PAMME with little or no melanin are usually difficult to diagnose endoscopically and pathologically and is often misdiagnosed as poorly differentiated carcinoma. This was true for our case because IHC did not reveal the presence of specific melanoma markers (MHB-45 and S-100) initially. Therefore, malignant melanoma should be considered when esophageal tumors with undetermined patterns of IHC staining are observed.

Radical surgery is reportedly the preferential treatment for PAMME in only a few cases when there was a curative intent. Five-year survival rate for radical surgery was 37% [6]. A Japanese study revealed that 1- and 5-year survival rates were 74.1 and 30.7%, respectively [10]. These data suggest that resectable PAMME has a relatively good prognosis. Conversely, the efficacy of palliative surgery was uncertain as mean survival after local resection of PAMME was only 9 months [8]. Therefore, alternative therapies to surgery should be considered for unresectable PAMME or its relapse. Immunotherapies and molecular targeted therapies have dramatically improved prognosis for cutaneous melanoma. Conversely, only one case has been reported where immunotherapy was used for PAMME; however, no study has reported the use of molecular targeted therapy. Rochefort *et al.* reported that nivolumab monotherapy against PAMME was well tolerated and progression-free survival was 5 months [11]. Genetic aberrations in PAMME were analyzed in two previous studies [12, 13], but only one study revealed BRAF mutation (V600E) in one out of 10 cases [12]. This shows that BRAF inhibitor and MEK inhibitor combination therapies may be effective in appropriate cases.

In conclusion, to our knowledge, this is the first report of PAMME treated with nivolumab and ipilimumab. We hope our report was able to present new insights into the treatment strategies for extracutaneous melanomas.
